# Sulfur Metabolism Actively Promotes Initiation of Cell Division in Yeast

**DOI:** 10.1371/journal.pone.0008018

**Published:** 2009-11-24

**Authors:** Heidi M. Blank, Shefali Gajjar, Andrey Belyanin, Michael Polymenis

**Affiliations:** Department of Biochemistry and Biophysics, Texas A&M University, College Station, Texas, United States of America; Auburn University, United States of America

## Abstract

**Background:**

Sulfur metabolism is required for initiation of cell division, but whether or not it can actively promote cell division remains unknown.

**Methodology/Principal Findings:**

Here we show that yeast cells with more mtDNA have an expanded reductive phase of their metabolic cycle and an increased sulfur metabolic flux. We also show that in wild type cells manipulations of sulfur metabolic flux phenocopy the enhanced growth rate of cells with more mtDNA. Furthermore, introduction of a hyperactive cystathionine-β-synthase (CBS) allele in wild type cells accelerates initiation of DNA replication.

**Conclusions/Significance:**

Our results reveal a novel connection between a key sulfur metabolic enzyme, CBS, and the cell cycle. Since the analogous hyperactive CBS allele in human CBS suppresses other disease-causing CBS mutations, our findings may be relevant for human pathology. Taken together, our results demonstrate the importance of sulfur metabolism in actively promoting initiation of cell division.

## Introduction

Growth is rate limiting for cell division. It has been known for some time that growth and metabolism are required for cell division to take place [1]. However, whether metabolism can actively promote cell division remains largely unaddressed due to the previous use of loss-of-function metabolic mutants when studying the coordination of growth and division. The *S. cerevisiae* nuclear gene *ABF2^+^* encodes a highly conserved mitochondrial DNA maintenance protein, called TFAM in animals, which binds to and bends mitochondrial DNA (mtDNA) [2], [3]. Moderate over-expression of Abf2p, from a low copy number plasmid or from two extra copies integrated in the genome, increases the amount of mtDNA by 50–150% [4]. We have previously shown that moderate over-expression of Abf2p causes cells to increase in size faster and accelerate initiation of DNA replication in the nucleus [5]. Furthermore, cells with more mtDNA proliferate faster than their wild type counterparts when cultured under carbon limiting conditions [5]. These unique properties of cells over-expressing Abf2p represent an experimental system that allows for the eventual dissection of how metabolism can promote cell division.

Sulfur metabolism is critical for many cellular processes, and sulfur metabolic flux correlates with growth rate in yeast [6], [7]. It has also been known for decades that yeast cells with disrupted sulfur metabolism arrest before initiation of DNA replication [8]. Homocysteine lies at a critical juncture between the trans-sulfuration pathway that leads to cysteine and glutathione (GSH) biosynthesis, and the branch that leads to S-adenosylmethionine (SAM) biosynthesis. Cystathionine-β-synthase (Cys4p in yeast, CBS in mammals) catalyzes pyridoxal 5′-phosphate-dependent (PLP) synthesis of cystathionine from serine and homocysteine [9]. In yeast, loss of Cys4p leads to cysteine auxotrophy [10], while even in rich media cell proliferation is delayed by ∼4-fold [11]. Among natural yeast isolates *CYS4* polymorphisms are the most common cause of sensitivity to pharmacological compounds [12]. In humans, mutations in CBS cause homocystinuria, associated with vascular disease [13], with >130 CBS mutations identified in patients [14]. Interestingly, a hyperactive CBS allele that encodes a truncated CBS suppresses other disease-causing mutations [15]. CBS activity positively correlates with cell proliferation in humans and yeast [16]. However, it is not known if CBS activity and sulfur metabolic flux are simply required for cell proliferation, or whether they can also actively promote initiation of cell division.

In this paper, we show that cells with more mtDNA, which have an accelerated growth rate, also have an increased sulfur metabolic flux. Furthermore, an up-regulation of sulfur metabolism is sufficient to trigger initiation of DNA replication in wild type cells. Finally, we show that in the presence of a hyperactive CBS allele alone, initiation of cell division is accelerated.

## Results and Discussion

### An Increase in mtDNA Levels Expands the Yeast Metabolic Cycle (YMC)

We had previously shown that cells moderately over-expressing Abf2p proliferate faster than their wild type counterparts [5]. Furthermore, we found that these cells accelerate initiation of DNA replication by increasing the rate of cell size increase, or their “growth rate” (see Fig. S1 and [5]). In contrast, most known accelerators reduce the “critical size” for division, and they have no effect on growth rate. Thus, we reasoned that cells over-expressing Abf2p likely carry “gain-of-function” metabolic alterations that promote cell cycle progression. We then used this strain as a tool to identify metabolic signatures that might impact on the timing of initiation of DNA replication.

To determine how metabolism differs in cells with more mtDNA, we first analyzed their metabolic cycle. When grown under carbon-limited steady-state conditions in a chemostat, yeast cells display robust oscillations in their oxygen consumption as they alternate between glycolytic and respiratory metabolism [17], [18], [19]. Following the nomenclature of Tu et al [17], the metabolic cycle can be divided into three major phases: oxidative (Ox), reductive/building (RB), and reductive/charging (RC). DNA replication is restricted to the reductive phases [19], [20]. This dual metabolic and cell cycle synchrony provides a unique experimental approach for studying the coupling of metabolism and cell division.

**Figure 1:**
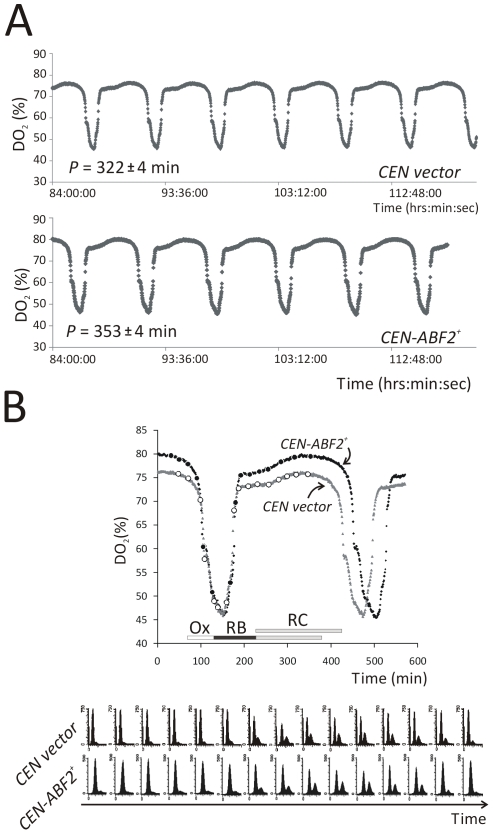
An increase in mtDNA levels expands the Yeast Metabolic Cycle (YMC). *A*, Oscillations of dissolved oxygen concentrations (% saturation, DO_2_) in continuous cultures of cells carrying either a control empty plasmid (*CEN vector*), or a plasmid containing *ABF2* (*CEN-ABF2^+^*). The average (± SD) period, *P*, calculated from the experiments shown is indicated. *B*, The metabolic cycles of the two indicated strains were superimposed and shown in higher resolution. At regular intervals as indicated by the circles (open for control, and filled for Abf2p over-expressing cells, respectively), samples were taken and analyzed for DNA content by flow cytometry (shown at the bottom). The corresponding oxidative (Ox), reductive/biosynthetic (RB), and reductive/charging (RC) phases, based on Tu et al [17], are shown.

We evaluated cells transformed with a low-copy centromeric plasmid containing *ABF2* (*CEN-ABF2^+^*) versus empty vector transformants (*CEN vector*). Cells with more mtDNA had a longer period of the metabolic cycle (5.9 hr vs. 5.3 hr), and specifically showed an expansion of the RC phase (Fig. 1). In both strains, the metabolic cycle gated cell division, and DNA replication was triggered only in the reductive phases of the metabolic cycles (Fig. 1B), as has been previously reported [17], [19], [21]. Although the period of the metabolic cycles of numerous mutants is shorter than wild type [20], an expansion of the metabolic cycle we observed in cells with more mtDNA is very rare [22].

### Sulfur Metabolic Flux Is Increased in Cells with More mtDNA

**Figure 2:**
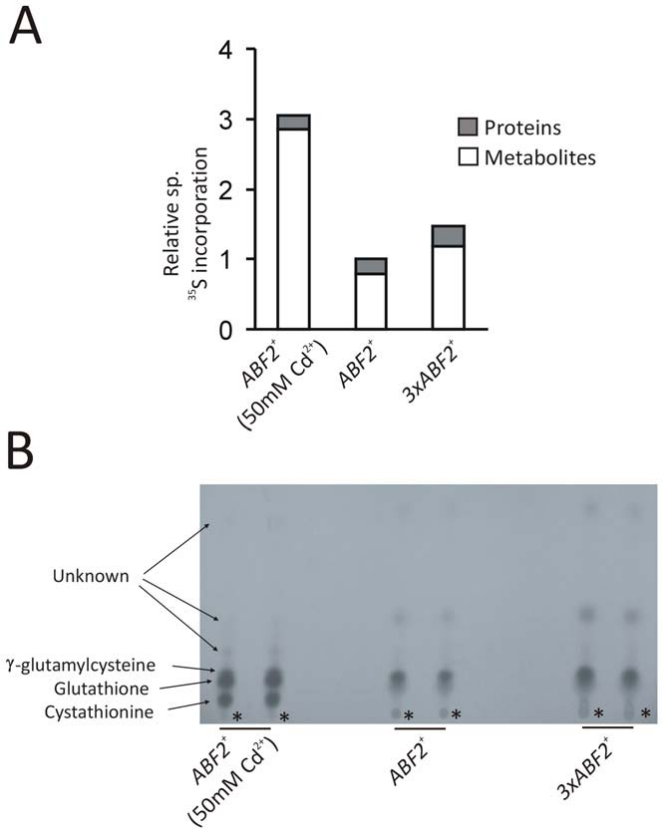
Sulfur metabolite levels are increased in cells with more mtDNA. *A*, Relative levels of ^35^S incorporation into proteins and metabolites from ^35^S -labeled cultures of the indicated strains. Incorporated radioactivity from each extract was quantified by liquid scintillation counting. Results shown are the average of two independent experiments. *B*, The metabolite fractions from (*A*) were resolved by cellulose thin-layer chromatography. Due to the oxidation with performic acid during sample preparation (see Methods), “glutathione” includes both the oxidized and reduced forms. The asterisks (*) at the lowest spots in all samples correspond to the origins, where the samples were applied on the TLC plate.

Sulfur metabolism is crucial to the metabolic cycle. Glutathione (GSH), the cell's main redox buffer, and other sulfur metabolites peak during the reductive phase [23], [24], the same phase that was expanded in cells over-expressing Abf2p. Because of these links between sulfur metabolism and the RC phase of the metabolic cycle, we sought to determine if sulfur metabolism was altered in cells over-expressing Abf2p. No rate-limiting step exists in the sulfur metabolic pathway in yeast, and a change in sulfur metabolite and/or enzyme levels should affect the flux of the overall pathway [25]. To examine if sulfur metabolite levels were altered in *3xABF2^+^* cells, exponentially growing cells in batch cultures were radiolabeled with inorganic ^35^S-labeled ammonium sulfate. Metabolites were water-extracted by boiling the cells and proteins were TCA-extracted from the cell pellet after removal of the metabolite fraction. As a control, we also treated wild type cells with Cd^2+^ sulfate, which is toxic. To cope with the toxicity, cells produce large amounts of glutathione to shuttle cadmium into the vacuoles, at the expense of protein synthesis [26]. As expected, overall sulfur metabolites were increased in these cells (Fig. 2A), with a reduction of radioactivity in the protein fraction. Importantly, sulfur metabolite levels were increased significantly (>40%) in *3xABF2^+^* cells versus wild type (*ABF2^+^*) cells, but this increase of sulfur metabolites was not at the expense of protein synthesis as in the Cd^2+^ treated cells (Fig. 2A). Metabolite fractions were further resolved by cellulose thin layer chromatography (TLC) after oxidation with performic acid. Sulfur metabolite levels, including total glutathione levels (reduced plus oxidized forms), were increased in cells with more mtDNA (Fig. 2B).

We also obtained a genome-wide gene expression profile from cells with increased mtDNA levels (Fig. S2). To minimize differences due to variability of batch culture conditions, the samples were collected from steady-state glucose-limited, but not metabolically oscillating, chemostats. Under these conditions, cells over-expressing *ABF2* proliferate faster and have altered cell cycle progression [5]. We observed the expected higher levels of *ABF2* due to the two extra chromosomal copies of *ABF2*, and also the increased expression from the rDNA (*RDN*) locus, which we had shown to be de-repressed in *3xABF2^+^* cells [5]. Otherwise, there were very few changes in the steady-state mRNA levels in *3xABF2^+^* cells (Fig. S2). In retrospect, this is not surprising. Previous microarray experiments identified only 34 genes whose expression was up-regulated (>2-fold) in ρ° petite cells (which do not have mtDNA) compared to ρ^+^ cells [27]. Interestingly, regarding mRNAs encoding sulfur metabolic enzymes, there were small (Fig. S3A), but concerted changes: the mRNA levels for most enzymes were slightly elevated, except for *STR2* and *STR3*, which encode enzymes (cystathionine-γ-synthase and cystathionine-β-lyase, respectively) that allow synthesis of homocysteine from cysteine away from glutathione. These expectations were also verified at the protein level (Fig. S3B). Thus, expression of sulfur metabolic enzymes from both the GSH and SAM branches is not reduced, and even perhaps slightly elevated. These results, together with the increased sulfur metabolite levels we found in cells with more mtDNA strongly suggest that overall sulfur metabolic flux is elevated, in accordance with the expansion of the RC phase of the metabolic cycle of these cells (Fig. 1). Our findings may have broader relevance, because over-expression of the animal ortholog of Abf2p, TFAM, has been recently shown to restore glucose secretion in a mouse model of maturity-onset diabetes [28], and protect from age-dependent impairment of brain performance [29].

### An Increase in Sulfur Metabolic Flux Accelerates Initiation of Cell Division

In yeast, sulfur metabolism is required for cell division to take place [8], and transcription of certain genes involved in sulfur metabolic pathways is cell cycle regulated, peaking at the onset of cell division [30]. Furthermore, our results that an increase in sulfur metabolic flux is associated with accelerated initiation of cell division in cells with more mtDNA is consistent with other data that sulfur metabolic flux correlates with growth rate [6]. However, all these results do not necessarily point to a *causal* role of sulfur metabolism in the control of cell division.

**Figure 3:**
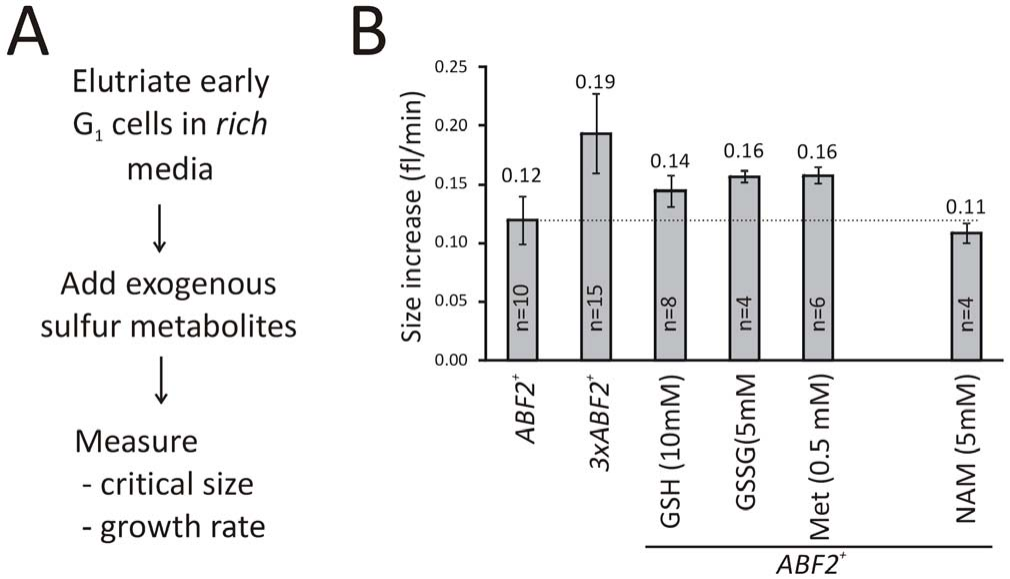
An increase in sulfur metabolic flux accelerates initiation of cell division. *A*, Schematic of our approach to measure cell cycle parameters after addition of exogenous metabolites. *B*, The rate of cell size increase (fl/min) of the indicated strains and metabolites added are shown. Values shown are the average (± SD) of the indicated number of experiments.

We reasoned that if the increase in sulfur metabolic flux we observed in cells with more mtDNA is responsible for the accelerated growth rate and initiation of cell division in these cells, then an increase in sulfur metabolic flux in wild type cells might lead to similar effects. It has been previously shown that exogenous addition of sulfur metabolites is sufficient to increase the flux of the overall pathway, because there is no rate-limiting step in the sulfur metabolic pathway in yeast [25]. Consequently, we then examined if adding exogenous sulfur metabolites to wild type cells would phenocopy the enhanced growth rate seen in cells over-expressing Abf2p. Cells were centrifugally elutriated to collect a synchronous population of small, un-budded cells in G1. Note that budding marks initiation of cell division, and serves as a convenient morphological landmark for the G1/S transition. Cells were then re-suspended in fresh, rich media containing extra sulfur metabolites and their cell cycle progression was monitored (Fig. 3A). The reported growth rates (Fig. 3B) are the summary of many repeats of individual experiments (Fig. S1). Remarkably, adding either reduced (GSH) or oxidized (GSSG) forms of glutathione significantly increased the growth rate of wild type cells, as did the addition of methionine. Adding additional GSH to *3xABF2^+^* cells did not further enhance their growth rate (not shown). We also tested the effect of nicotinamide (NAM) because it inhibits NAD^+^-dependent enzymes, including the deacetylase Sir2p [31], [32]. Sir2p and similar enzymes are thought to couple energy status with many cellular processes, and we have previously shown that Sir2p acts antagonistically to Abf2p [5]. Interestingly, however, NAM did not alter the rate of cell size increase (Fig. 3B). Overall, although the rich media (YPD) used in this experiment were thought to set an upper limit to how fast cells can grow, addition of sulfur metabolites further increased growth rate.

In all the above experiments we also measured the cell size at which cells trigger a new round of cell division (Fig. S1). We did not find significant cell size decreases that could explain the accelerated initiation of cell division (Fig. S1). Thus, from a detailed analysis of both “growth rate” and “critical size” we conclude that an up-regulation of sulfur metabolic flux in wild type cells is sufficient to significantly accelerate initiation of cell division, which can be largely attributed to an increase in growth rate. This is a surprising and important finding, because there is no reason to expect that the growth rate of a fast-proliferating unicellular eukaryote grown in rich medium that is not limited for any particular nutrient, including sulfur, can be further increased by up-regulating sulfur metabolic flux.

### Cystathionine-β-synthase and Cell Cycle Progression

Among several sulfur metabolic enzymes examined, only reducing the expression of *CYS4*, the gene encoding cystathionine β-synthase, abolished the yeast metabolic cycles [23]. Consequently, we decided to study the effects of cystathionine β-synthase perturbations on cell cycle progression. It was known that in the absence of Cys4p cell proliferation is delayed by ∼4-fold [11], but we asked the opposite question: Can Cys4p positively affect cell division? To answer this we needed a gain-of-function *CYS4* allele. The C-terminal domain (∼145 amino acid residues) is thought to participate in protein-protein interactions [33], and this domain interferes with catalytic activity, because its truncation elevates activity in the human [15] and yeast [34] enzymes. We confirmed these observations by expressing and purifying the full-length (Cys4p) and the truncated (Cys4p(1–353)) yeast enzymes from bacteria, and comparing their kinetic properties (Fig. S4). The truncated Cys4p(1–353) is a more efficient enzyme, with a *k*_cat_/*K*_M_ ∼3-fold higher than that of the full-length enzyme. We then generated a strain that carried only a truncated Cys4p(1–353) instead of the full-length Cys4p, and examined cell cycle progression from synchronous cultures as described above (Figs. S1 and 4).

**Figure 4:**
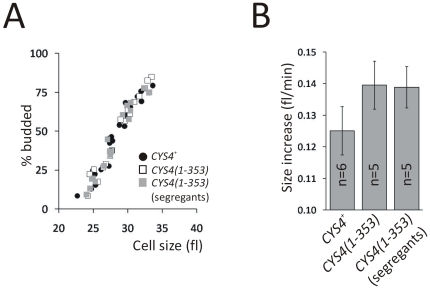
Cys4p(1–353) accelerates initiation of cell division. *A*, The “critical size” for division is unchanged in *CYS4(1–353)* cells. The raw data points showing the percent of budded cells as a function of cell size, from separate independent elutriation experiments with *CYS4^+^* and *CYS4(1–353)* strains. The data points shown were from the linear portion of each experiment, when the percentage of budded cells began to increase. *B*, The rate of cell size increase is elevated in *CYS4(1–353)* cells. The rate of cell size increase was determined from the same elutriation experiments shown in (*A*), as described in Fig. S1. The raw data for these calculations are shown in Fig. S5.

We found that *CYS4(1–353)* cells have a shorter G1 than their otherwise isogenic wild type (*CYS4^+^*) counterparts (Fig. 4). *CYS4(1–353)* cells bud at the same size (28.9±0.2 fl, n = 5) as (*CYS4^+^*) cells (28.6±0.5 fl, n = 6; *P* = 0.25, based on a 2-tailed Student's *t* test) (Fig. 4A). However, *CYS4(1–353)* cells increased in size faster (0.14±0.01 fl/min, n = 5) than *CYS4^+^* cells (0.12±0.01 fl/min, n = 6; *P* = 0.01 based on a 2-tailed Student's *t* test) (Figs. 4B and S5). Thus, even though *CYS4(1–353)* cells do not bud at a smaller size, they reach that size ∼15% faster than their *CYS4^+^* counterparts. To exclude the possibility of secondary mutations contributing to the phenotype, we mated the original mutant haploid with the isogenic wild type but of the opposing mating type. The resulting diploid heterozygotes were then sporulated and dissected, to segregate out any secondary mutations. The growth rate and critical size were then measured in detail by elutriation. We obtained essential identical values as before, demonstrating that the accelerated growth rate of these cells is due to the *CYS4(1–353)* allele (Fig. 4). In conclusion, introduction of a single hypermorphic allele of a sulfur metabolic enzyme, cystathionine-β-synthase, is sufficient to accelerate growth rate and initiation of cell division (Fig. 4). Together with our sulfur metabolite analysis, these results reveal a critical role of sulfur metabolism in actively controlling cell division. Perhaps the nature of sulfur metabolism, lacking any rate-limiting steps, allows for adjustments in the overall flux that cannot be easily achieved in other pathways.

Finally, although the most common cause of human disorders in sulfur metabolism is mutations in CBS [13], [14], a role for CBS in cell cycle progression in humans has not been examined. There is a very high degree of conservation between Cys4p and human CBS. Importantly, human CBS can complement yeast cells lacking Cys4p [35]. Thus, findings from our yeast studies might be relevant to studies of the human CBS ortholog and sulfur metabolism in general.

## Materials and Methods

### Strains and Plasmids

The haploid *3xABF2^+^* strain and its wild type counterpart (14ww) have been described elsewhere [4]. For the yeast metabolic oscillations we used the CEN.PK strain described in [17]. From this strain we generated a Ura^−^ auxotroph (strain SCMSP202) by plating on solid media containing 5-Fluoroorotic Acid (5-FOA). This strain was then transformed with a *URA3*-marked plasmid containing *ABF2* (*CEN-ABF2^+^*) [4], [36], or the empty vector (*CEN vector*). To introduce a *CYS4(1–353)-13MYC::KANMX* allele in the standard BY4742 (*MATα; his3Δ; leu2Δ; lys2Δ; ura3Δ*) strain, we followed a single-step PCR replacement protocol as described [37], to generate strain SCMSP179. The corresponding wild-type isogenic strain carrying a full-length *CYS4-13MYC::KANMX* allele was also constructed (strain SCMSP178). Both strains were genotyped by PCR and tested for protein expression of the corresponding alleles. The TAP-tagged strains used in Fig. S3B were purchased from Open Biosystems.

For recombinant expression of Cys4p and Cys4p(1–353) as N-terminal 6xHis fusions, DNA fragments encoding these proteins were generated by PCR and inserted into the NheI and XhoI sites of pET-28 (Novagen), to allow for T7-driven protein expression in *E.coli*. The proteins of interest were purified using TALON® Co^2+^ beads according to the manufacturer's instructions (Clontech), and analyzed for their kinetic parameters as described in [38].

### Yeast Cultivation and Cell Cycle Analysis

For batch cultures we followed established yeast protocols [39]. Conditions for yeast metabolic oscillations in chemostat cultures were identical to previously published methods [17]. Centrifugal elutriation conditions, DNA content, “growth rate” and “critical size” analyses were done as we have described [5]. For the metabolites used in Fig. 3, Glutathione (reduced and oxidized) was from USB (Cleveland, OH), L-methionine from Avocado Research Chemicals (Heysham, UK), and Nicotinamide from Sigma (St. Louis, MO).

### Metabolite Levels and Cellulose TLC

^35^S metabolic labeling was done for 4 hr, as in [26], using 1.8×10^7^ cells in each case. Cadmium treated cells were supplemented with 50 mM Cd^2+^ sulfate for one hour prior to labeling. Metabolites were extracted and separated by TLC according to [40], with the exception that cells were quenched with cycloheximide (50 µg/ml) and sodium azide (0.1%) just prior to harvesting.

### Microarray Analysis

We prepared total RNA from *ABF2^+^* and *3xABF2^+^* cells grown in chemostats under glucose limitation at a dilution rate of 0.2 h^−1^, as we have described [5]. These were not metabolically oscillating cultures. The Microarray Core Facility of University of Texas Southwestern Medical Center performed the microarray analysis. The data from this experiment is MIAME compliant and the raw data has been deposited in ArrayExpress (Accession Code: E-MEXP-2362). The *ABF2^+^* and *3xABF2^+^* signals were first normalized against the signals for TFIID (*YER148W*), *SRB4* (*YER022W*), *WBP1*(*YEL002C*) and *TDH1* (*YJL052W*), which were linear in both datasets, taking the average for each control and averaging those ratios. We then calculated the log_2_(*3xABF2^+^/ABF2^+^*) values shown in Figs. S2 and S3A.

### Immunoblot Analysis

Immunoblots were done as described earlier [5]. To detect the TAP epitope we used the PAP reagent (Sigma), while for Cdk detection we used an anti-PSTAIR antibody (Abcam), according to their recommendations.

## Supporting Information

Figure S1 A, The rate of cell size increase for each elutriation experiment of the indicated strains and metabolite treatments is shown. From these graphs we determined the rate of size increase reported in Fig. 3, calculated as described in [1]. B, Graphs showing the percent of budded cells as a function of cell size, from separate independent elutriation experiments with the indicated strains and metabolite treatments. The data points shown were from the linear portion of each experiment, when the percentage of budded cells began to increase, and used to determine the critical size for division, as described in [1]. The average (± SD) is shown in each case. The graphs shown in (A) and (B) for the ABF2+ and 3xABF2+ strains (without added metabolites), incorporate not only new experiments, but also earlier ones we had described in [1].
(0.64 MB TIF)

Figure S2 Microarray expression profile of cells with more mtDNA (3xABF2+) vs. wild type (ABF2+). The genes that showed more than a 2-fold change in their steady-state mRNA levels between the two strains are indicated. In addition to ABF2 and the RDN locus, which were up-regulated in 3xABF2+ cells as expected, there were some additional changes that were anticipated. For example, the increased transcription of SNZ1 and SNO1 in wild type (ABF2+) cells was expected because expression of these genes is increased in strains auxotrophic for tryptophan and uracil, such as the wild-type (ABF2+) strain. The 3xABF2+ strain is not auxotrophic for tryptophan and uracil. Furthermore, up-regulation of SFC1 is consistent with up-regulation of mitochondrial processes in 3xABF2+ cells, because SFC1 encodes a mitochondrial transporter, which transports succinate into and fumarate out of the mitochondrion.
(0.42 MB TIF)

Figure S3 A. Sulfur metabolic pathways and expression profile in cells with more mtDNA (3xABF2+) vs. wild type (ABF2+). The genes encoding enzymes with increased expression are shown in red, while those with reduced expression are shown in green, while for one gene (GSH2), there was no change. The numbers above each gene indicate the corresponding log2(3xABF2+/ABF2+) values. B. To test expression at the protein level, we used strains (purchased from Open Biosystems) carrying a TAP-tagged allele of the enzyme of interest under the control of the endogenous promoter, and compared the steady-state protein levels in cells transformed either with empty CEN-vector, or with CEN-ABF2+. We examined expression in two different dilutions of the cell extract in question, 1∶1 (left panels) and 1∶10 (right panels), and in each case the samples were loaded in triplicate. We show the levels of Ecm38p and Str3p, which were over- and under-expressed, respectively, in the microarray experiment (see Fig. S3A). As a loading control, we show the levels of Cdk in the same samples.
(0.76 MB TIF)

Figure S4 Kinetic parameters of recombinant Cys4p and Cys4p(1–353). Rate-dependence of the yeast full-length Cys4p and Cys4p(1–353) enzymes on the concentration of cystathionine is shown. Cystathionine-β-synthase activity was measured using a spectrophotometric assay [2], where the reverse-physiological hydrolysis of L-cystathionine to L-Ser and L-homocysteine was detected as absorbance changes at 412 nm, through the reaction of 5,5′-dithiobis-(2-nitrobenzoic acid) (DTNB) with the free thiol of the L-homocysteine product. The enzymes displayed strong substrate inhibition above 0.4 mM, and this was accounted for when we plotted the data using the non-linear regression function of KaleidaGraph (Synergy Software) to obtain the average (± SEM) kinetic parameters shown, from at least four independent experiments in each case.
(0.57 MB TIF)

Figure S5 The rate of cell size increase for each elutriation experiment of the CYS4+, CYS4(1–353) and the segregant CYS4(1–353) cells is shown. From these graphs we determined the rates reported in Fig. 4, calculated as described in [1].
(0.87 MB TIF)

## References

[1] Pringle JR, Hartwell LH, Strathern JD, Broach JR (1981). The Saccharomyces cerevisiae cell cycle.. The molecular biology of the yeast Saccharomyces.

[2] Stigter D (2004). Packaging of single DNA molecules by the yeast mitochondrial protein Abf2p: reinterpretation of recent single molecule experiments.. Biophys Chem.

[3] Friddle RW, Klare JE, Martin SS, Corzett M, Balhorn R (2004). Mechanism of DNA compaction by yeast mitochondrial protein Abf2p.. Biophys J.

[4] Zelenaya-Troitskaya O, Newman SM, Okamoto K, Perlman PS, Butow RA (1998). Functions of the high mobility group protein, Abf2p, in mitochondrial DNA segregation, recombination and copy number in Saccharomyces cerevisiae.. Genetics.

[5] Blank HM, Li C, Mueller JE, Bogomolnaya LM, Bryk M (2008). An increase in mitochondrial DNA promotes nuclear DNA replication in yeast.. PLoS Genet.

[6] Castrillo JI, Zeef LA, Hoyle DC, Zhang N, Hayes A (2007). Growth control of the eukaryote cell: a systems biology study in yeast.. J Biol.

[7] Brosnan JT, Brosnan ME (2006). The sulfur-containing amino acids: an overview.. J Nutr.

[8] Unger MW, Hartwell LH (1976). Control of cell division in Saccharomyces cerevisiae by methionyl-tRNA.. Proc Natl Acad Sci U S A.

[9] Banerjee R, Evande R, Kabil O, Ojha S, Taoka S (2003). Reaction mechanism and regulation of cystathionine beta-synthase.. Biochim Biophys Acta.

[10] Thomas D, Surdin-Kerjan Y (1997). Metabolism of sulfur amino acids in Saccharomyces cerevisiae.. Microbiol Mol Biol Rev.

[11] Giaever G, Chu AM, Ni L, Connelly C, Riles L (2002). Functional profiling of the *Saccharomyces cerevisiae* genome.. Nature.

[12] Kim HS, Fay JC (2007). Genetic variation in the cysteine biosynthesis pathway causes sensitivity to pharmacological compounds.. Proc Natl Acad Sci U S A.

[13] Fowler B (2005). Homocysteine: overview of biochemistry, molecular biology, and role in disease processes.. Semin Vasc Med.

[14] Kraus JP, Janosik M, Kozich V, Mandell R, Shih V (1999). Cystathionine beta-synthase mutations in homocystinuria.. Hum Mutat.

[15] Shan X, Kruger WD (1998). Correction of disease-causing CBS mutations in yeast.. Nat Genet.

[16] Maclean KN, Janosik M, Kraus E, Kozich V, Allen RH (2002). Cystathionine beta-synthase is coordinately regulated with proliferation through a redox-sensitive mechanism in cultured human cells and Saccharomyces cerevisiae.. J Cell Physiol.

[17] Tu BP, Kudlicki A, Rowicka M, McKnight SL (2005). Logic of the yeast metabolic cycle: temporal compartmentalization of cellular processes.. Science.

[18] Richard P (2003). The rhythm of yeast.. FEMS Microbiol Rev.

[19] Klevecz RR, Bolen J, Forrest G, Murray DB (2004). A genomewide oscillation in transcription gates DNA replication and cell cycle.. Proc Natl Acad Sci U S A.

[20] Chen Z, Odstrcil EA, Tu BP, McKnight SL (2007). Restriction of DNA replication to the reductive phase of the metabolic cycle protects genome integrity.. Science.

[21] Porro D, Martegani E, Ranzi BM, Alberghina L (1988). Oscillations in continuous cultures of yeast: A segragated parameter analysis.. Biotechnol Bioengin.

[22] Tu BP, McKnight SL (2009). Evidence of carbon monoxide-mediated phase advancement of the yeast metabolic cycle.. Proc Natl Acad Sci U S A.

[23] Tu BP, Mohler RE, Liu JC, Dombek KM, Young ET (2007). Cyclic changes in metabolic state during the life of a yeast cell.. Proc Natl Acad Sci U S A.

[24] Murray DB, Beckmann M, Kitano H (2007). Regulation of yeast oscillatory dynamics.. Proc Natl Acad Sci U S A.

[25] Lafaye A, Junot C, Pereira Y, Lagniel G, Tabet JC (2005). Combined proteome and metabolite-profiling analyses reveal surprising insights into yeast sulfur metabolism.. J Biol Chem.

[26] Fauchon M, Lagniel G, Aude JC, Lombardia L, Soularue P (2002). Sulfur sparing in the yeast proteome in response to sulfur demand.. Mol Cell.

[27] Epstein CB, Waddle JA, Hale Wt, Dave V, Thornton J (2001). Genome-wide responses to mitochondrial dysfunction.. Mol Biol Cell.

[28] Gauthier BR, Wiederkehr A, Baquie M, Dai C, Powers AC (2009). PDX1 deficiency causes mitochondrial dysfunction and defective insulin secretion through TFAM suppression.. Cell Metab.

[29] Hayashi Y, Yoshida M, Yamato M, Ide T, Wu Z (2008). Reverse of age-dependent memory impairment and mitochondrial DNA damage in microglia by an overexpression of human mitochondrial transcription factor a in mice.. J Neurosci.

[30] Spellman PT, Sherlock G, Zhang MQ, Iyer VR, Anders K (1998). Comprehensive identification of cell cycle-regulated genes of the yeast *Saccharomyces cerevisiae* by microarray hybridization.. Mol Biol Cell.

[31] Landry J, Slama JT, Sternglanz R (2000). Role of NAD(+) in the deacetylase activity of the SIR2-like proteins.. Biochem Biophys Res Commun.

[32] Bitterman KJ, Anderson RM, Cohen HY, Latorre-Esteves M, Sinclair DA (2002). Inhibition of silencing and accelerated aging by nicotinamide, a putative negative regulator of yeast sir2 and human SIRT1.. J Biol Chem.

[33] Kabil O, Zhou Y, Banerjee R (2006). Human cystathionine beta-synthase is a target for sumoylation.. Biochemistry.

[34] Jhee KH, McPhie P, Miles EW (2000). Domain architecture of the heme-independent yeast cystathionine beta-synthase provides insights into mechanisms of catalysis and regulation.. Biochemistry.

[35] Kruger WD, Cox DR (1994). A yeast system for expression of human cystathionine beta-synthase: structural and functional conservation of the human and yeast genes.. Proc Natl Acad Sci U S A.

[36] O'Rourke TW, Doudican NA, Mackereth MD, Doetsch PW, Shadel GS (2002). Mitochondrial dysfunction due to oxidative mitochondrial DNA damage is reduced through cooperative actions of diverse proteins.. Mol Cell Biol.

[37] Longtine MS, McKenzie A, Demarini DJ, Shah NG, Wach A (1998). Additional modules for versatile and economical PCR-based gene deletion and modification in *Saccharomyces cerevisiae*.. Yeast.

[38] Belew MS, Quazi FI, Willmore WG, Aitken SM (2009). Kinetic characterization of recombinant human cystathionine beta-synthase purified from E. coli.. Protein Expr Purif.

[39] Kaiser C, Michaelis S, Mitchell A (1994). Methods in Yeast Genetics..

[40] Vido K, Spector D, Lagniel G, Lopez S, Toledano MB (2001). A proteome analysis of the cadmium response in Saccharomyces cerevisiae.. J Biol Chem.

