# Supplementary figures and images for: Sulfur Metabolism Actively Promotes Initiation of Cell Division in Yeast
Source: PLoS One. 2009 Nov 24;4(11):e8018. doi: 10.1371/journal.pone.0008018 (PMC2776973; doi:10.1371/journal.pone.0008018)

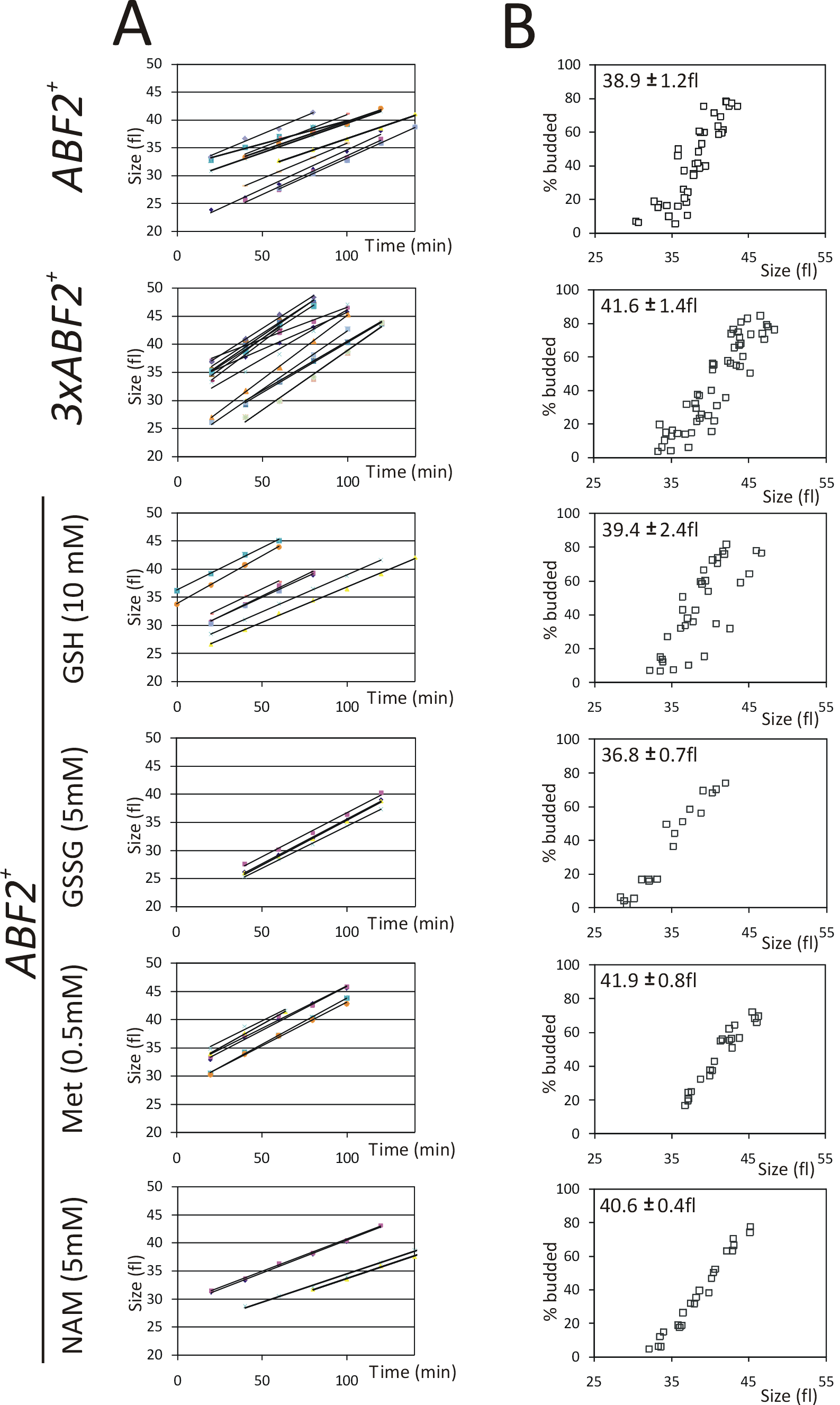

Supplement: Figure S1 — A, The rate of cell size increase for each elutriation experiment of the indicated strains and metabolite treatments is shown. From these graphs we determined the rate of size increase reported in Fig. 3, calculated as described in [1]. B, Graphs showing the percent of budded cells as a function of cell size, from separate independent elutriation experiments with the indicated strains and metabolite treatments. The data points shown were from the linear portion of each experiment, when the percentage of budded cells began to increase, and used to determine the critical size for division, as described in [1]. The average (± SD) is shown in each case. The graphs shown in (A) and (B) for the ABF2+ and 3xABF2+ strains (without added metabolites), incorporate not only new experiments, but also earlier ones we had described in [1]. (0.64 MB TIF) [file pone.0008018.s001.tif]

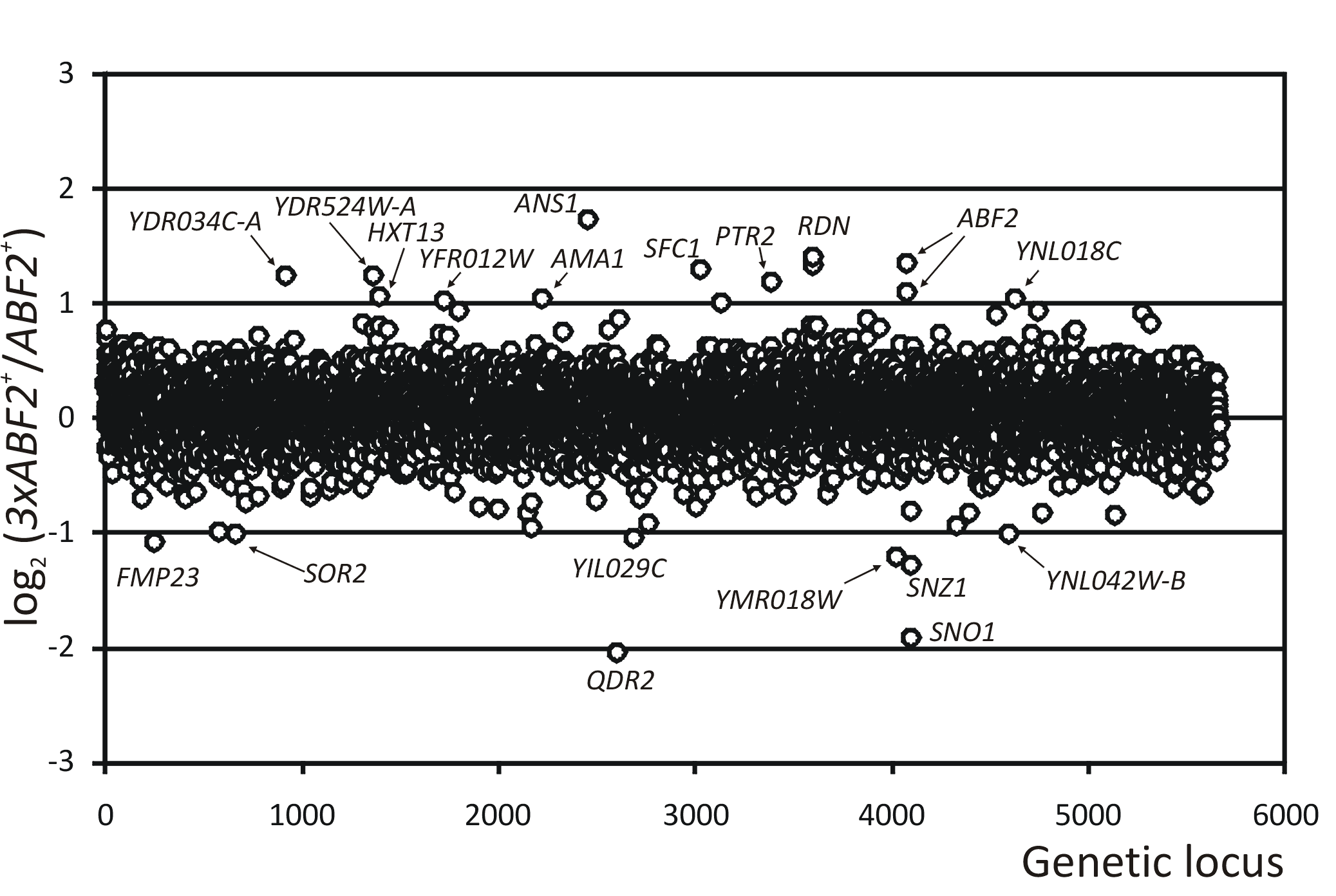

Supplement: Figure S2 — Microarray expression profile of cells with more mtDNA (3xABF2+) vs. wild type (ABF2+). The genes that showed more than a 2-fold change in their steady-state mRNA levels between the two strains are indicated. In addition to ABF2 and the RDN locus, which were up-regulated in 3xABF2+ cells as expected, there were some additional changes that were anticipated. For example, the increased transcription of SNZ1 and SNO1 in wild type (ABF2+) cells was expected because expression of these genes is increased in strains auxotrophic for tryptophan and uracil, such as the wild-type (ABF2+) strain. The 3xABF2+ strain is not auxotrophic for tryptophan and uracil. Furthermore, up-regulation of SFC1 is consistent with up-regulation of mitochondrial processes in 3xABF2+ cells, because SFC1 encodes a mitochondrial transporter, which transports succinate into and fumarate out of the mitochondrion. (0.42 MB TIF) [file pone.0008018.s002.tif]

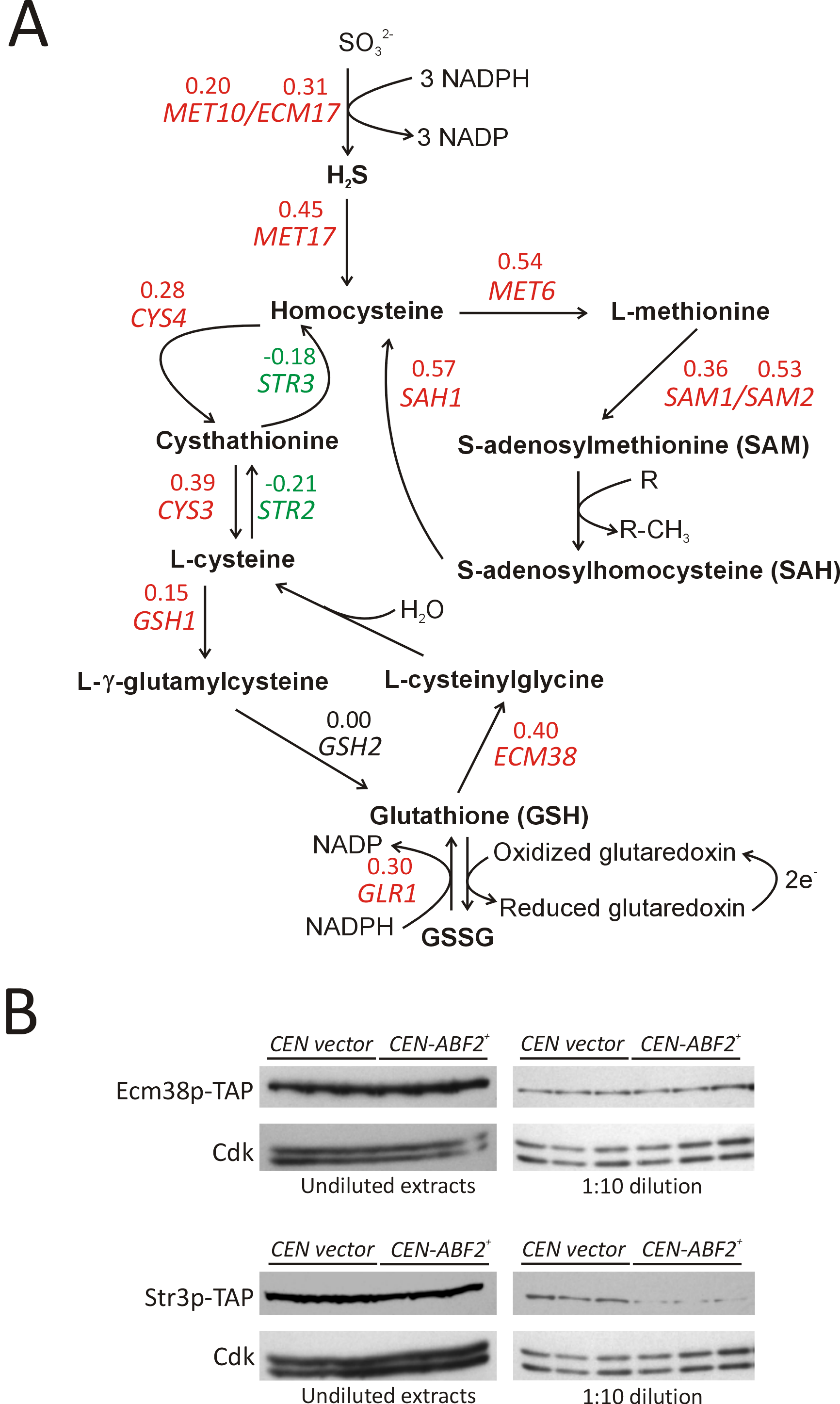

Supplement: Figure S3 — A. Sulfur metabolic pathways and expression profile in cells with more mtDNA (3xABF2+) vs. wild type (ABF2+). The genes encoding enzymes with increased expression are shown in red, while those with reduced expression are shown in green, while for one gene (GSH2), there was no change. The numbers above each gene indicate the corresponding log2(3xABF2+/ABF2+) values. B. To test expression at the protein level, we used strains (purchased from Open Biosystems) carrying a TAP-tagged allele of the enzyme of interest under the control of the endogenous promoter, and compared the steady-state protein levels in cells transformed either with empty CEN-vector, or with CEN-ABF2+. We examined expression in two different dilutions of the cell extract in question, 1∶1 (left panels) and 1∶10 (right panels), and in each case the samples were loaded in triplicate. We show the levels of Ecm38p and Str3p, which were over- and under-expressed, respectively, in the microarray experiment (see Fig. S3A). As a loading control, we show the levels of Cdk in the same samples. (0.76 MB TIF) [file pone.0008018.s003.tif]

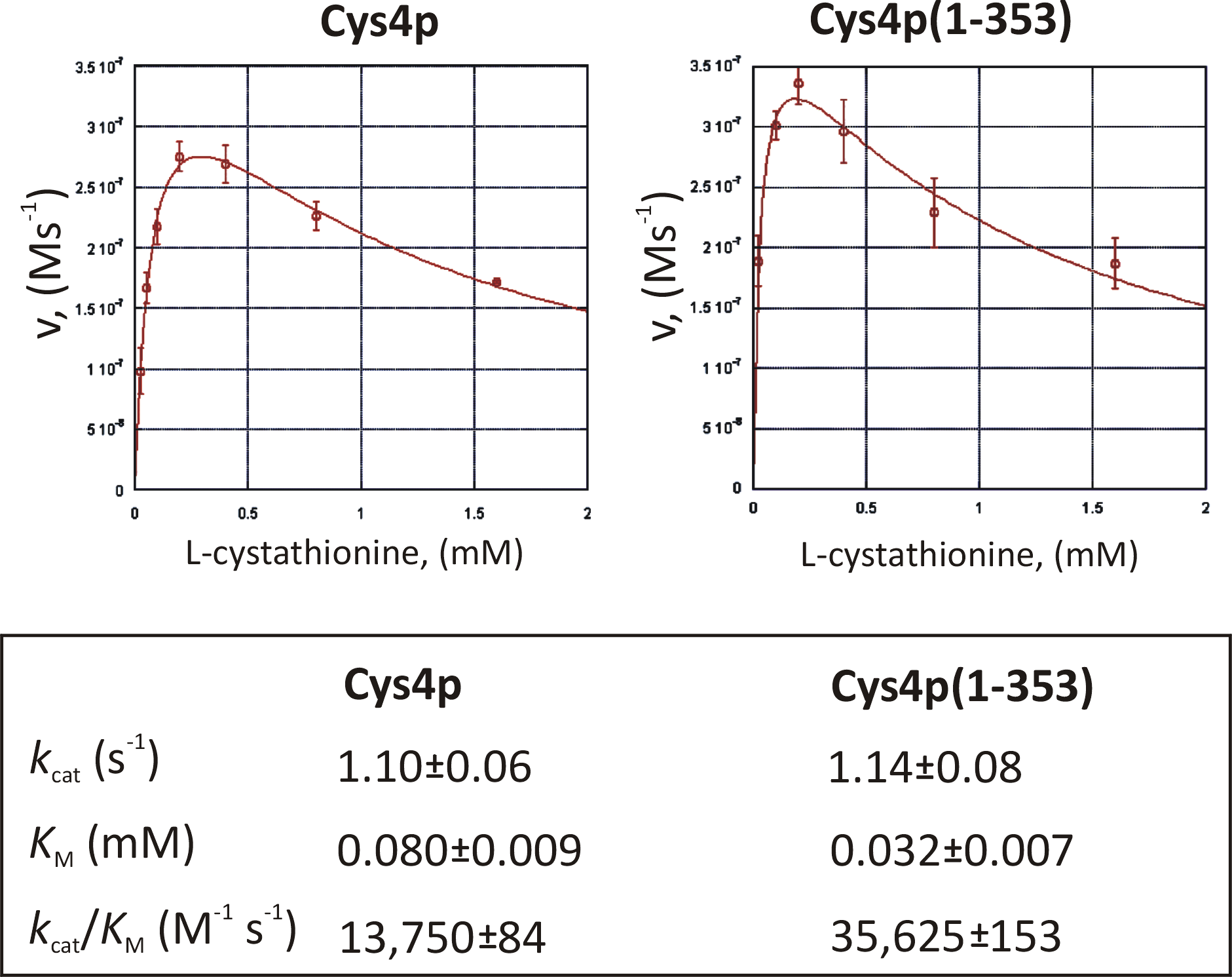

Supplement: Figure S4 — Kinetic parameters of recombinant Cys4p and Cys4p(1–353). Rate-dependence of the yeast full-length Cys4p and Cys4p(1–353) enzymes on the concentration of cystathionine is shown. Cystathionine-β-synthase activity was measured using a spectrophotometric assay [2], where the reverse-physiological hydrolysis of L-cystathionine to L-Ser and L-homocysteine was detected as absorbance changes at 412 nm, through the reaction of 5,5′-dithiobis-(2-nitrobenzoic acid) (DTNB) with the free thiol of the L-homocysteine product. The enzymes displayed strong substrate inhibition above 0.4 mM, and this was accounted for when we plotted the data using the non-linear regression function of KaleidaGraph (Synergy Software) to obtain the average (± SEM) kinetic parameters shown, from at least four independent experiments in each case. (0.57 MB TIF) [file pone.0008018.s004.tif]

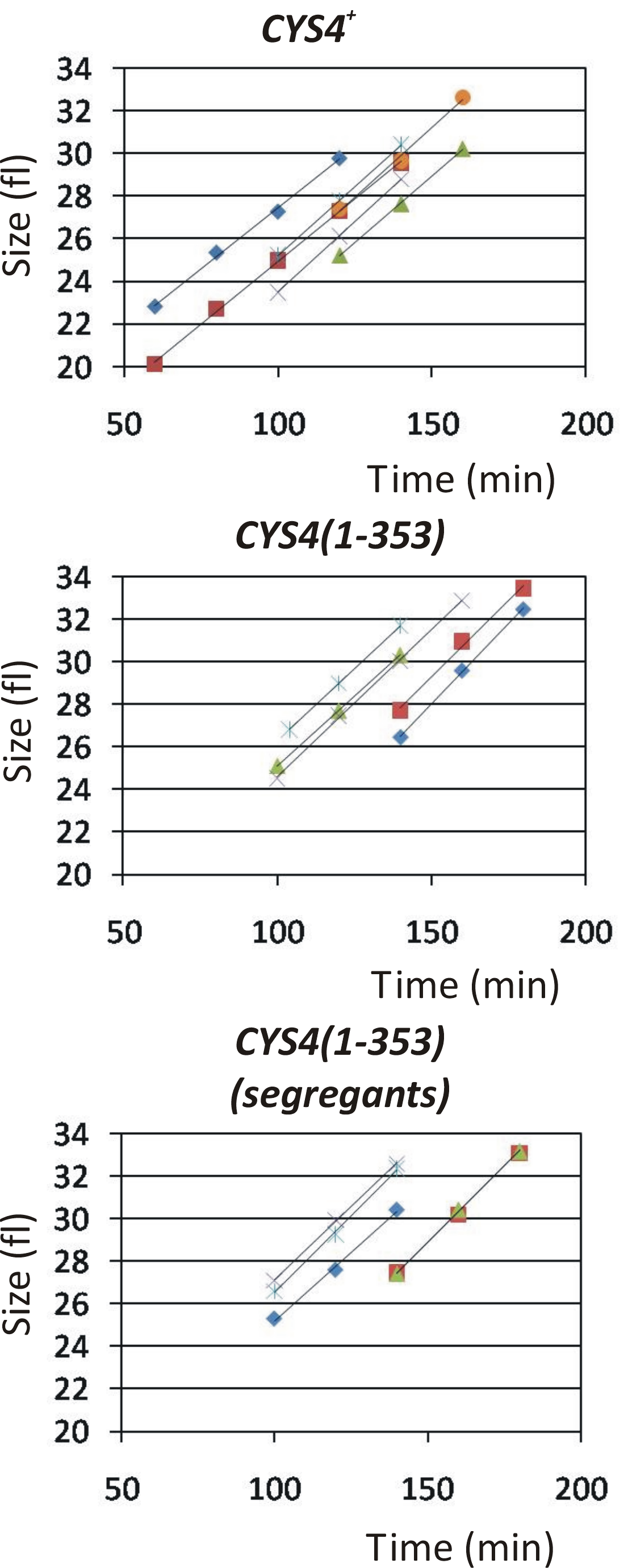

Supplement: Figure S5 — The rate of cell size increase for each elutriation experiment of the CYS4+, CYS4(1–353) and the segregant CYS4(1–353) cells is shown. From these graphs we determined the rates reported in Fig. 4, calculated as described in [1]. (0.87 MB TIF) [file pone.0008018.s005.tif]
